# Health care workers causing large nosocomial outbreaks: a systematic review

**DOI:** 10.1186/1471-2334-13-98

**Published:** 2013-02-22

**Authors:** Lisa Danzmann, Petra Gastmeier, Frank Schwab, Ralf-Peter Vonberg

**Affiliations:** 1 Institute for Medical Microbiology and Hospital Epidemiology, Hannover Medical School, Carl-Neuberg-Str. 1, Hannover D-30625, Germany; 2 Institute for Hygiene and Environmental Health, Charité – University Medicine Berlin, Berlin, Germany

**Keywords:** Nosocomial outbreak, Staff, Personnel, Health care workers, Health care associated infection

## Abstract

**Backgrounds:**

Staff in the hospital itself may be the source of a nosocomial outbreak (NO). But the role of undetected carriers as an outbreak source is yet unknown.

**Methods:**

A systematic review was conducted to evaluate outbreaks caused by health care workers (HCW). The Worldwide Outbreak Database and PubMed served as primary sources of data. Articles in English, German or French were included. Other reviews were excluded. There were no restrictions with respect to the date of publication.

Data on setting, pathogens, route of transmission, and characteristics of the HCW was retrieved. Data from large outbreaks were compared to smaller outbreaks.

**Results:**

152 outbreaks were included, mainly from surgery, neonatology, and gynecology departments. Most frequent corresponding infections were surgical site infections, infection by hepatitis B virus, and septicemia. Hepatitis B virus (27 NO), *S. aureus* (49 NO) and *S. pyogenes* (19 NO) were the predominant pathogens involved. 59 outbreaks (41.5%) derived from physicians and 56 outbreaks (39.4%) derived from nurses. Transmission mainly occurred via direct contact. Surgical and pediatric departments were significantly associated with smaller outbreaks, and gynecology with larger outbreaks. Awareness of carrier status significantly decreased the risk of causing large outbreaks.

**Conclusions:**

As NO caused by HCW represent a rare event, screening of personnel should not be performed regularly. However, if certain species of microorganisms are involved, the possibility of a carrier should be taken into account.

## Background

Healthcare associated infections (HCAI) are infections that are acquired as a result of healthcare interventions. Most HCAI occur sporadically only. However, epidemics of HCAI may also take place and are than called a nosocomial outbreak (NO). NO always represent extremely frightening incidents, but may still affect any medical department at any time in principle. Once a NO has been recognized as such, an outbreak investigation is usually initiated in order to discover its source. Doing so, the outbreak’s source may get traced to a particular single health care worker (HCW) in some of the cases. If now a HCW, in fact, happens to be the most likely or proven cause of pathogen spread to patients and nosocomial infections, all of a sudden several uncertainties and questions arise, for example: Was the HCW aware of his/her carrier status? Were occupational physicians of the hospital wrong at evaluating the risk of transmission from a colonized or infected HCW? Are there obvious breaches in infection control measures? Are there specific risk factors that may have contributed to an extraordinary high transmission rate?

There are several reports of NO that started from a HCW. However, every report by itself is very much influenced by the local situation. Thus, one can hardly generalize the experiences from a single NO description. Only a systematic evaluation of a large number of outbreak reports will provide a less biased assessment of data.

This article presents findings of a systematic analysis of all kinds of published NO that were caused by HCW. By this, we will provide HCW characteristics and factors that may dramatically facilitate pathogen spread.

## Methods

### Retrieval strategy

We conducted a systematic review of the medical literature. Search of the literature was performed on June 23^rd^ 2010. In order to find appropriate descriptions of NO we first of all made a data request for outbreaks whose source was “personnel” in the Outbreak Database (http://www.outbreak-database.com). This is an internet-based, worldwide database for NO continuously updated by the Institute for Hygiene and Environmental Medicine, Charité – University Medicine Berlin (Germany) currently containing over 2,900 outbreaks [1]. In a second step we supplemented our data by a PubMed search on September 3^rd^ 2010, consisting of the following search terms [(“nosocomial”) AND (“outbreak” OR “epidemic”) AND (“personnel” OR “staff” OR “health care worker”)]. Finally, we looked through the references of the articles which had been included by then to complete the search.

### Exclusion and inclusion criteria

Besides staff in the hospital being the outbreak’s source, inclusion criteria were as follows: articles written in English, German or French language. Reviews were excluded to avoid bias. There were no restrictions with respect to the date of publication.

### Extracted data

(a) We extracted data on the setting (time, country, medical department), patients (type of infections, causative agent, number of fatal cases), (b) characteristics about the staff that caused the outbreak (kind of profession, infection among themselves vs. colonization only, route of transmission, awareness of their own positive carriage status of pathogen, compliance to hand hygiene, length of work experience), and (c) the infection control measures implemented to terminate the outbreak (screening of patients and/or staff, disinfection and sterilization procedures, isolation of patients, application of antimicrobial substances, education of personnel, use of protective clothing, improvement in hand hygiene, closure of ward, sampling of medical devices and/or the environment, changes in patient-staff ratio, use of vaccinations, no implementing of any infection control measures at all). Data was primarily extracted by one author (L.D.) and than independently cross-checked by another author (R.P.V.). A third author (P.G.) got involved in the case of disagreement between the other two authors.

### Large outbreaks

NO were defined as large outbreaks (LO), when the number of affected patients was equal or greater than the median number of patients in all NO.

### Statistics

In the descriptive statistic, rates were calculated for LO stratified by the following risk factors: Type of department, age groups (neonates, infants, children, adolescents, adults, seniors), type of ward (intensive care, peripheral ward, outpatients clinic, operating theatre), outbreak happening before or after 1989 (to identify the trend of the past years and as this was the time when vaccination against hepatitis B virus (HBV) was already recommended in several countries, e. g. the USA [2,3]), causative agent, specific characteristics of the source, and infection control measures. For univariate analysis (contingency tables), the Fisher exact test was used, with a significance level of 0.05. Multiple logistic regression analysis was performed with stepwise variable selection to detect LO with the parameters mentioned above. We set p ≤ 0.05 for entering a parameter into the model. We used the commercial statistical package Statistical Analysis System (SAS, Version 9.2, the SAS Institute Inc., Cary, NC, USA) for the analysis. Significance level was set at 0.05.

## Results

### Included articles

**Figure 1:**
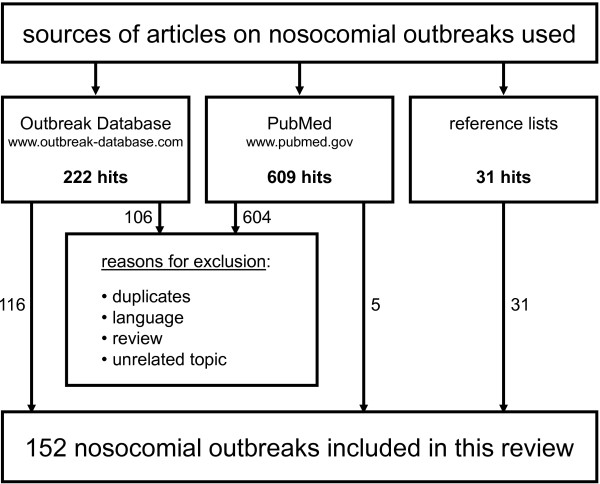
Retrieval of articles.

At the time point of retrieval the Outbreak Database comprised 222 articles in which HCW were filed as the outbreak’s source. 116 descriptions thereof met the inclusion criteria. Outbreaks in facilities other than hospitals (e.g., nursing homes) got excluded, as did NO with a HCW being suspected only (but not proven) the true source. Altogether 5% of all outbreaks published in the Outbreak Database were included. The PubMed search (609 hits) finally came up with 5 more NO reports. 31 additional reports were found via the search of reference lists. Thus, this systematic review is based on overall 152 NO descriptions caused by HCWs (Figure 1). The complete list of articles can be retrieved as an Additional file 1. It consists of 140 articles (9 authors described 2 NO and another author described 4 different NO within one single article).

### Univariate analysis

#### Setting

The 152 NO took place in 26 countries, mainly in the US (67 NO), the UK (29 NO), and France (8 NO). The time frame of occurrence was 1958 through 2006. NO lasted between 1 and 287 weeks (mean: 28.4; median: 10.5). 9 NO showed a polyphasic progress with more than one peak in the epidemic curve.

#### Patients

Overall, 1,449 patients (thereof at least 51 fatal cases) were affected ranging from 1 to 75 patients per NO (mean: 9.5; median: 7.0). Thus, a number of patients ≥ 7 divided 76 large outbreaks (LO) from 76 other smaller NO.

#### Medical departments and type of ward

As shown in Table 1, departments of surgery (76 NO), neonatology (41 NO), gynecology (20 NO), pediatrics (9 NO), and internal medicine (2 NO) were most often affected by NO derived from HCW. LO were less often noticed in surgical departments (42.1%) compared to other types of medical departments (59.1%). The same applies to pediatric departments where the proportion of LO was 11.1% only vs. 53.2% in other medical fields. In 2 NO no specific medical department was mentioned.

**Table 1:** Risk factors for the occurrence of large outbreaks (≥ 7 patients) as determined by univariate analysis (more than one type may be affected)

**Risk factor**	**Risk factor present**			**Risk factor lacking**			**p-value**
	**# LO**	**# all NO**	**%**	**# LO**	**# all NO**	**%**	
departments (n=150)*							
surgery	32	76	42.1	44	74	59.1	0.036
neonatology	24	41	58.5	52	109	47.7	0.274
gynecology	14	20	70.0	62	130	47.7	0.091
pediatrics	1	9	11.1	75	141	53.2	0.017
internal medicine	1	2	50.0	75	148	50.7	1.000
other	11	21	52.4	65	129	50.4	1.000
type of ward (n=150)*							
operating theatre	26	63	41.3	50	87	57.5	0.068
peripheral ward	33	59	55.9	43	91	47.3	0.320
intensive care unit	17	28	60.7	59	122	48.4	0.296
outpatient clinic	4	9	44.4	72	141	51.1	0.744
age groups (n=147)*							
neonates (≤ 1 m)	24	39	61.5	52	108	48.1	0.191
infants (1 m - 1 y)	3	6	50.0	73	141	51.8	1.000
children (> 1–12 y)	4	14	28.6	72	133	54.1	0.092
adolescents (13–17 y)	9	13	69.2	67	134	50.0	0.249
adults (18–69 y)	49	93	52.7	27	54	50.0	0.864
seniors (≥ 70 y)	13	26	50.0	63	121	52.1	1.000
**microorganism (n=152)**							
bacteria	58	108	53.7	19	44	43.2	0.284
viruses	14	34	41.2	63	118	53.4	0.245
fungi	5	10	50.0	72	142	50.7	1.000
**species (n=152)**							
*S. aureus*	26	49	53.1	51	103	49.5	0.730
hepatitis B virus	9	27	33.3	68	125	54.4	0.057
*S. pyogenes*	12	19	63.2	65	133	48.9	0.328
*Candida spp.*	5	8	62.5	72	144	50.0	0.719
*P. aeruginosa*	2	7	28.6	75	145	51.7	0.273
**transmission (n=152)**							
contact	53	105	50.5	24	47	51.1	1.000
droplets	8	17	47.1	69	135	51.1	0.801
airborne	11	16	68.8	66	136	48.5	0.860
foodborne	3	6	50.0	74	146	50.7	1.000
unknown	3	13	23.1	74	139	53.2	0.045
**HCW characteristics**							
colonization only	43	73	58.9	34	79	43.0	0.054
HWC infected	31	70	44.3	46	82	56.1	0.193
blood borne infection	9	27	33.3	68	125	54.4	0.057
aware of carrier status	3	14	21.4	62	118	52.5	0.045
proper HH compliance	12	21	57.1	9	16	56.3	1.000
work experience > 5 y	9	18	50.0	8	12	66.7	0.536

*LO* = large outbreak (≥ 7 patients affected); *NO* = nosocomial outbreak; *HCW* = health care worker; *HH* = hand hygiene; *more than one type may be affected.

Transmission of most NO occurred in operating theatres (63 NO) and on peripheral wards (59 NO). The risk of LO via operating theatres was slightly decreased (41.3% vs. 57.5%; p = 0.068) but failed to reach statistical significance.

#### Infection types

Besides several cases of colonization, there were also at least 960 documented cases of nosocomial infections among the 1,449 patients. The most frequent nosocomial infections were surgical site infections (SSI; 256), HBV infections (212), septicemia (67), gastroenteritis (42), hepatitis C virus infections (HCV) (21), urinary tract infections (20), and meningitis (13).

#### Causative agent

Transmission of bacteria occurred in 108 of the 152 NO. Viral spread (34 NO) and fungi (10 NO) were less often the causative agents. Table 1 shows a detailed distribution of the most frequently detected microorganisms in NO caused by HCW which were *S. aureus* (49 NO), HBV (27 NO), and Group A streptococci (19 NO). Regardless of the number of patients (LO vs. other NO), spread of the pathogen via direct contact was the main route of transmission, followed by droplets and airborne transmission. Although the source was known (HCW), the specific way of transmission still remained unknown in 8.6% of all NO.

#### Characteristics of index personnel

**Figure 2:**
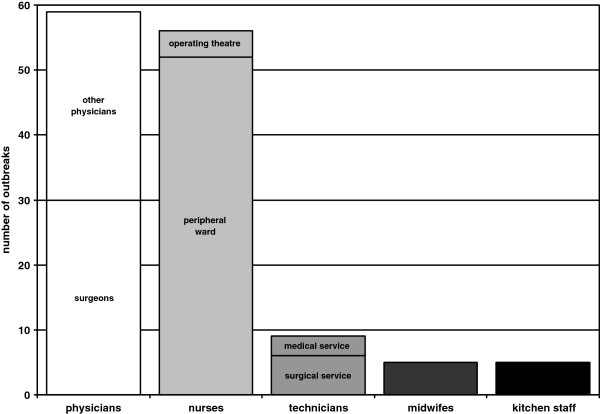
Occupation of health care workers causing nosocomial outbreaks.

Despite their rather low proportion among hospital staff, physicians were the group of professionals who caused most outbreaks (59 NO [41.5%], thereof 30 NO caused by surgeons) compared to 56 NO (39.4%) caused by nurses (Figure 2). Other professions frequently involved were technical staff (9 NO), kitchen staff (5 NO), and midwives (5 NO).

A total of 73 of the spreading HCWs were colonized only and 70 others were themselves infected by the pathogen (as described in the outbreak reports) before spread to their patients took place (Table 1). If the index HCW was colonized only (but not infected) large outbreaks were more common (58.9%) compared to outbreaks where the index person also showed signs and symptoms of infection (43.0%). Especially HCW suffering from blood-borne infections were less likely to become a super spreader (33.3% vs. 54.4%; Table 1).

Data on work experience was available for few outbreak events only. However, a subgroup analysis of HCW, stratified by the time of employment, revealed that work experience of a HCW was neither a predicting nor a protecting factor for being a super spreader later on. In 18 NO (50.0%) the HCW had worked for less than 5 years compared to 15 NO (41.7%) in which the index HCW had work experience for at least 10 years.

Precise information about the spreading HCW with respect to hand hygiene (HH) was also scarce. Only 37 authors provided information on the previous HH compliance of the spreading HCW, thereof HH compliance was considered “adequate” in 21 of 37 NO (56.8%) but “insufficient” in 16 NO (43.2%).

Of the 152 spreading HCW only 14 declared that they had previously been aware of their positive pathogen carrier status; 10 of these 14 HCW were affected by some kind of viral hepatitis. However, the HCW’s awareness of harboring the pathogen significantly lowered the risk of subsequently causing large outbreaks (21.4% vs. 52.5%; p = 0.045).

#### Infection control measures

**Figure 3:**
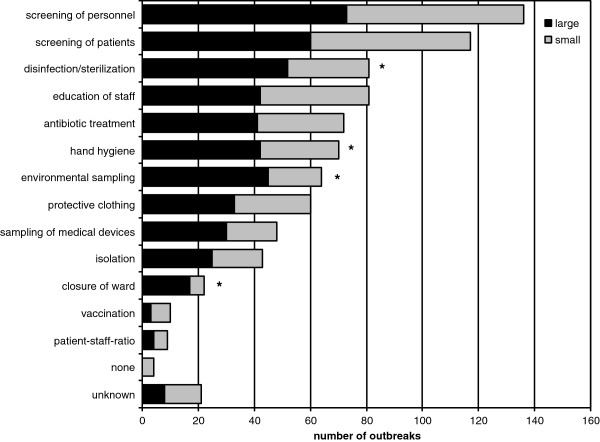
Distribution of infection control measures initiated in large and small outbreaks (* = significant difference).

In order to provide a complete overview on outbreaks caused by HCW, corresponding measures were also recorded in this systematic review. Screening for pathogen carriage were the measures carried out most frequently as seen in Figure 3. Furthermore, there were several infection control measures that were more often performed in larger outbreaks such as changes in disinfection and/or sterilization processes (52 vs. 19 NO), enforcement of hand hygiene compliance (42 vs. 28 NO), environmental sampling (45 vs. 19 NO), and closure of an entire ward/unit (17 vs. 5 NO).

### Multiple logistic regression analysis

In order to identify independent factors that increase the risk of a LO, a multiple logistic regression analysis was performed as described above. The remaining characteristics after stepwise variable selection are shown in Table 2.

**Table 2:** Independent risk factors for the occurrence of large outbreaks (≥ 7 patients) as determined by multiple logistic regression analysis with stepwise variable selection

**Risk factor**	**# all NO**	**Odds ratio**	**95% confidence interval**	**p-value**
**departments**				
surgery	76	0.42	0.19–0.92	0.030
gynecology	20	6.89	1.55–30.63	0.011
pediatrics	9	0.05	0.00–0.45	0.008
**occurrence after 1989**				
reference value: no	71	1.00	n. d.	0.024
yes	72	0.48	0.22–1.06	0.068
**aware of carrier status**				
reference value: no	116	1.00	n. d.	0.024
yes	14	0.11	0.02–0.55	0.008
**transmission**				
unknown	13	0.16	0.03–0.75	0.020

*NO* = nosocomial outbreak; *n. d.* = not defined.

HCW in gynecological departments caused significantly more often large outbreaks with an odds ratio (OR) = 6.89, in contrast to surgical departments (OR = 0.42) and pediatrics (OR = 0.05) where large outbreaks were much less common. In addition, awareness of the HCW which is carrier of an infectious agent significantly reduced the risk (by 89%) of causing a LO. In the last 20 years there was some tendency towards smaller outbreaks.

## Discussion

### NO in surgery

Most of all included NO caused by staff happened in surgical departments (76 of 150 NO), but the univariate and the multiple regression analysis specified these outbreaks to be significantly smaller than the other NO. Surgeons may extraordinary strong focus on nosocomial infections, because those represent the most frequent complication in their medical field. Thus, spreaders may be detected earlier which then will lead to smaller NO. The operating theatre was the location of transmission in 80% (61 of 76 NO) of all NO in surgery. An explanation may be that there is a very short distance between head and hands of the surgeon and the patient, who is rather vulnerable during invasive procedures. 142 of 411 infections transmitted during operations (130 HBV, 10 HCV and 2 HIV) were blood borne. Surgeons often suffer from small undetected excoriations or injuries caused by sharp bone fragments, needles and other instruments. Transmission may occur through minimal lesions in gloves [4]. Harpaz et al. [5] described an NO where a surgeon infected 19 patients with HBV, but the exact route of transmission remained unclear. He had performed an adequate operating technique and no obvious breaches in hygiene could be discovered. Other common agents were gram positive bacteria like *Streptococcus* Group A (121 infections) or *S. aureus* (101 infections). These agents caused mostly SSI (87.8%; 195 of 222 infections).

### NO in gynecology

NO in gynecology are not only very common (20 NO; thereof 11 NO in obstetrics), they are also significantly larger (OR = 6.89) than NO in other medical departments (Table 2). In 7 NO a gynecologist transmitted HBV to a patient during an operation. And in the remaining 2 NO kitchen workers contaminated food with enteritic *Salmonella* and infected patients in the whole hospital. In obstetrics the most common agent was *S. aureus* (5 of 11 NO), which was being transmitted to neonates and parturient. There were also other agents transmitted via contact (*Streptococcus* group A in 4 NO and *S. marcescens* in 1 NO). During a delivery the women giving birth have close and prolonged contact with e.g. midwifes (4 NO), which facilitates to transmit agents via contact. Dave et al. [6] reported 2 NO where midwifes transmitted *S. aureus* to 12 and 10 parturients and caused staphylococcal scalded skin syndrome (SSSS). The latest NO in obstetrics was an NO in France described by Occelli et al. [7] where an auxiliary nurse was nasal colonized with *S. aureus* and caused *bullous impetigo* in 7 neonates and colonization in 3 further neonates. Perhaps the tendency to early discharging of patients after birth may contribute to the problem of large NO in gynecology, because infections may occur only after discharge and therefore NO maybe detected later.

### HCW characteristics

The only significant detail we know about the spreading HCW is that those HCW who were aware of their positive carrier status caused smaller outbreaks than the median (Tables 1 and 2). There were 14 HCW that caused NO, although they knew of their infectivity, 12 physicians, 1 nurse and 1 acupuncturist. Thereof 10 employees who had a viral hepatitis (9 HBV, 1 HCV).

Being a carrier and knowing so may improve compliance to general infection control measures and by this lower the risk of pathogen spread. This result also suggests the presumption that HCW pay more attention to a possible NO, if they know of their risk to infect their patients. They detect NO earlier and therefore can terminate it earlier with less affected patients.

There is also the possibility that HCW denied awareness of their carrier status, because they could fear of job-related and judicial consequences, but a viral hepatitis is an infection that is probably more difficult to deny. In addition, viral hepatitis represents an infection, which is not easily transmitted and so NO may have been smaller. There is a tendency of blood borne infections to smaller NO, which is with a p = 0.57 nearly significant (Table 1).

HCW that were only colonized and not infected caused rather large NO (58.9%) than others (43.0%) (p = 0.54) (Table 1). Only 1.4% of the colonized HCW were aware of their carrier status, compared to 17.1% of infected HCW. HCW with more than 5 years of work experience caused LO just as often as HCW with 5 or less years of work experience did (Table 1). Experience of HCW does not necessarily provide significant more safety in patient care. In fact, there is a tendency of becoming less thorough in the compliance to recommendations [8].

Figure 2 shows that physicians caused most NO in absolute numbers (59 of 144 NO). Compared to the rather small proportion of physicians at all hospital staff, one may wonder why this group of HCW has likely been involved in NO. One explanation may be, that physicians have often and intensive contact, for example during invasive procedures, where the patient is rather vulnerable. Nearly all NO caused by physicians (72.9%; 43 of 59 NO) were transmitted in the operating theatre. NO caused by physicians may also get publicized more frequently than NO due to other HCW. Finally we cannot exclude the possibility of inadequate HH by physicians [9].

### General limitations

The most important limitation distorting these results is the publication bias. Presumably NO will have been detected more often than they get published. Supposedly those NO, that have been published have more often been of certain interest or came up with significant results.

Furthermore we could only include those outbreaks where the route of transmission could clearly be found out. Therefore easily transmittable agents like *Norovirus* and others are surely underrepresented. Many hospitals may have restrained from publishing their NO because of fear of bad reputation.

Another limitation is incomplete information in articles. For example we cannot provide data on the extent of cooperation between ward staff and infection control personnel, microbiologists, occupational health physicians or public health colleagues, but its quality often has a significant impact on both the prevention and the management of NO. There is also a lack of data on most socio-economic factors and/or underlying diseases of the patients that may predispose for a nosocomial infection. Especially information concerning the spreading HCW (e.g., on his HBV vaccination status) was scarce and complicated this analysis. The limit of 7 patients (= median) for LO is rather high, but reflects that chances of publication outrange chances of outbreak detection.

## Conclusion

*Practical consequences*. Screening of personnel should not be performed regularly, as less than 10% of NO are caused by HCW. However, if certain species of microorganisms (e.g. *S. aureus*, HBV, *S. pyogenes*) are involved, the possibility of a carrier should be taken into account.

## Competing interests

The authors herewith declare that there are no competing interests.

## Authors’ contributions

LD was responsible for collecting and interpreting of the data on the nosocomial outbreaks. PG has made substantial contributions to conception and design and revised the manuscript critically. FS performed statistical analyses. RPV cross-checked data extraction, participated in the conception an design of the study and primarily drafted the manuscript. All authors read and approved the final manuscript.

## Pre-publication history

The pre-publication history for this paper can be accessed here:

http://www.biomedcentral.com/1471-2334/13/98/prepub

## Supplementary Material

Additional file 1 Complete list of included articles. Click here for file
