# Supplementary material for: Health care workers causing large nosocomial outbreaks: a systematic review
Source: BMC Infect Dis. 2013 Feb 22;13:98. doi: 10.1186/1471-2334-13-98 (PMC3599984; doi:10.1186/1471-2334-13-98)
Supplement: Additional file 1 — Complete list of included articles. [file 1471-2334-13-98-S1.doc]

1. Alexander EM, Travis S, Booms C, Kaiser A, Fry NK, Harrison TG, Ganpot B, Klein JL: **Pertussis outbreak on a neonatal unit: identification of a healthcare worker as the likely source.** *J Hosp Infect* 2008, **69:**131-134.

2. Allen KD, Anson JJ, Parsons LA, Frost NG: **Staff carriage of methicillin-resistant Staphylococcus aureus (EMRSA 15) and the home environment: a case report.** *J Hosp Infect* 1997, **35:**307-311.

3. Ankers HE, Klapper PE, Cleator GM, Bailey AS, Tullo AB: **The role of a rapid diagnostic test (adenovirus immune dot-blot) in the control of an outbreak of adenovirus type 8 keratoconjunctivitis.** *Eye (Lond)* 1993, **7 (Pt 3 Suppl ):**15-17.

4. Anonymous: **Hospital outbreak of streptococcal wound infection--Utah.** *MMWR* 1976, **25:**141-142.

5. Anonymous: **Acute hepatitis B associated with gynaecological surgery.** *Lancet* 1980, **1:**1-6.

6. Anonymous: **Acute hepatitis B following gynaecological surgery.** *J Hosp Infect* 1987, **9:**34-38.

7. Anonymous: **From the Centers for Disease Control. Foodborne nosocomial outbreak of Salmonella.** *JAMA* 1991, **266:**3405-3406.

8. Anonymous: **Transmission of hepatitis B to patients from four infected surgeons without hepatitis B e antigen. The Incident Investigation Teams and others.** *N Engl J Med* 1997, **336:**178-184.

9. Anonymous: **Nosocomial group A streptococcal infections associated with asymptomatic health-care workers--Maryland and California, 1997.** *MMWR Morb Mortal Wkly Rep* 1999, **48:**163-166.

10. Anonymous: **An outbreak of hepatitis B associated with reusable subdermal electroencephalogram electrodes. Hepatitis B Outbreak Investigation Team.** *CMAJ* 2000, **162:**1127-1131.

11. Anonymous: **Mycobacterium tuberculosis transmission in a newborn nursery and maternity ward--New York City, 2003.** *MMWR Morb Mortal Wkly Rep* 2005, **54:**1280-1283.

12. Anonymous: **Outbreaks of pertussis associated with hospitals--Kentucky, Pennsylvania, and Oregon, 2003.** *MMWR Morb Mortal Wkly Rep* 2005, **54:**67-71.

13. Anonymous: **Hospital-acquired pertussis among newborns--Texas, 2004.** *MMWR Morb Mortal Wkly Rep* 2008, **57:**600-603.

14. Anthony BF, Giuliano DM, Oh W: **Nursery outbreak of staphylococcal scalded skin syndrome. Rapid identification of the epidemic bacterial strain.** *Am J Dis Child* 1972, **124:**41-44.

15. Ayliffe GA, Collins BJ: **Wound infections acquired from a disperser of an unusual strain of Staphylococcus aureus.** *J Clin Pathol* 1967, **20:**195-198.

16. Back NA, Linnemann CC, Jr., Pfaller MA, Staneck JL, Morthland V: **Recurrent epidemics caused by a single strain of erythromycin-resistant Staphylococcus aureus. The importance of molecular epidemiology.** *JAMA* 1993, **270:**1329-1333.

17. Barchiesi F, Caggiano G, Falconi DF, Montagna MT, Barbuti S, Scalise G: **Outbreak of fungemia due to Candida parapsilosis in a pediatric oncology unit.** *Diagn Microbiol Infect Dis* 2004, **49:**269-271.

18. Beck-Sague CM, Chong WH, Roy C, Anderson R, Jarvis WR: **Outbreak of surgical wound infections associated with total hip arthroplasty.** *Infect Control Hosp Epidemiol* 1992, **13:**526-534.

19. Belani A, Sherertz RJ, Sullivan ML, Russell BA, Reumen PD: **Outbreak of staphylococcal infection in two hospital nurseries traced to a single nasal carrier.** *Infect Control* 1986, **7:**487-490.

20. Ben Ayed S, Boutiba-Ben Boubaker I, Boukadida J, Hammami S, Ben Redjeb S: **Hospital acquired outbreak of methicillin-resistant Staphylococcus aureus infection initiated by a health care worker.** *Tunis Med* 2010, **88:**199-202.

21. Berkelman RL, Martin D, Graham DR, Mowry J, Freisem R, Weber JA, Ho JL, Allen JR: **Streptococcal wound infections caused by a vaginal carrier.** *JAMA* 1982, **247:**2680-2682.

22. Bertin ML, Vinski J, Schmitt S, Sabella C, Danziger-Isakov L, McHugh M, Procop GW, Hall G, Gordon SM, Goldfarb J: **Outbreak of methicillin-resistant Staphylococcus aureus colonization and infection in a neonatal intensive care unit epidemiologically linked to a healthcare worker with chronic otitis.** *Infect Control Hosp Epidemiol* 2006, **27:**581-585.

23. Betremieux P, Chevrier S, Quindos G, Sullivan D, Polonelli L, Guiguen C: **Use of DNA fingerprinting and biotyping methods to study a Candida albicans outbreak in a neonatal intensive care unit.** *Pediatr Infect Dis J* 1994, **13:**899-905.

24. Blanchard A, Ferris S, Chamaret S, Guetard D, Montagnier L: **Molecular evidence for nosocomial transmission of human immunodeficiency virus from a surgeon to one of his patients.** *J Virol* 1998, **72:**4537-4540.

25. Boszczowski I, Nicoletti C, Puccini DM, Pinheiro M, Soares RE, Van dH, I, Costa SF, Barone AA, Levin AS: **Outbreak of extended spectrum beta-lactamase-producing Klebsiella pneumoniae infection in a neonatal intensive care unit related to onychomycosis in a health care worker.** *Pediatr Infect Dis J* 2005, **24:**648-650.

26. Bouvet A, Fournier JM, Audurier A, Branger C, Orsoni A, Girard C: **Epidemiological markers for epidemic strain and carrier isolates in an outbreak of nosocomial oxacillin-resistant Staphylococcus aureus.** *J Clin Microbiol* 1990, **28:**1338-1341.

27. Boyce JM, Opal SM, Potter-Bynoe G, Medeiros AA: **Spread of methicillin-resistant Staphylococcus aureus in a hospital after exposure to a health care worker with chronic sinusitis.** *Clin Infect Dis* 1993, **17:**496-504.

28. Boyce JM, Potter-Bynoe G, Opal SM, Dziobek L, Medeiros AA: **A common-source outbreak of Staphylococcus epidermidis infections among patients undergoing cardiac surgery.** *J Infect Dis* 1990, **161:**493-499.

29. Bryant KA, Humbaugh K, Brothers K, Wright J, Pascual FB, Moran J, Murphy TV: **Measures to control an outbreak of pertussis in a neonatal intermediate care nursery after exposure to a healthcare worker.** *Infect Control Hosp Epidemiol* 2006, **27:**541-545.

30. Buch NA, Dhananjiya A: **A nursery outbreak of multidrug resistant Salmonella typhimurium.** *Indian Pediatr* 1998, **35:**455-459.

31. Burke JP, Ingall D, Klein JO, Gezon HM, Finland M: **Proteus mirabilis infections in a hospital nursery traced to a human carrier.** *N Engl J Med* 1971, **284:**115-121.

32. Carl M, Blakey DL, Francis DP, Maynard JE: **Interruption of hepatitis B transmission by modification of a gynaecologist's surgical technique.** *Lancet* 1982, **1:**731-733.

33. Carnicer-Pont D, White D, Pike C, Lyons M: **Influenza A outbreak in a community hospital in south east Wales, February 2005.** *Euro Surveill* 2005, **10:**E050217.

34. Casewell MW, Dalton MT, Webster M, Phillips I: **Gentamicin-resistant Klebsiella aerogenes in a urological ward.** *Lancet* 1977, **2:**444-446.

35. Cassettari VC, da Silveira IR, Dropa M, Lincopan N, Mamizuka EM, Matte MH, Matte GR, Menezes PR: **Risk factors for colonisation of newborn infants during an outbreak of extended-spectrum beta-lactamase-producing Klebsiella pneumoniae in an intermediate-risk neonatal unit.** *J Hosp Infect* 2009, **71:**340-347.

36. Ceyhan M, Yildirim I, Tekeli A, Yurdakok M, Us E, Altun B, Kutluk T, Cengiz AB, Gurbuz V, Barin C, Bagdat A, Cetinkaya D, Gur D, Tuncel O: **A Chryseobacterium meningosepticum outbreak observed in 3 clusters involving both neonatal and non-neonatal pediatric patients.** *Am J Infect Control* 2008, **36:**453-457.

37. Coovadia YM, Bhana RH, Johnson AP, Haffejee I, Marples RR: **A laboratory-confirmed outbreak of rifampicin-methicillin resistant Staphylococcus aureus (RMRSA) in a newborn nursery.** *J Hosp Infect* 1989, **14:**303-312.

38. Coutinho RA, Albrecht-van Lent P, Stoutjesdijk L, Meerburg-Snarenberg P, Courouce-Pauty AM, van Dijk BA, Kloek J: **Hepatitis B from doctors.** *Lancet* 1982, **1:**345-346.

39. Dave J, Reith S, Nash JQ, Marples RR, Dulake C: **A double outbreak of exfoliative toxin-producing strains of Staphylococcus aureus in a maternity unit.** *Epidemiol Infect* 1994, **112:**103-114.

40. David MD, Kearns AM, Gossain S, Ganner M, Holmes A: **Community-associated meticillin-resistant Staphylococcus aureus: nosocomial transmission in a neonatal unit.** *J Hosp Infect* 2006, **64:**244-250.

41. Dawson C, Darrell R: **Infections due to adenovirus type 8 in the United States. I. An outbreak of epidemic keratoconjunctivitis originating in a physician's office.** *N Engl J Med* 1963, **268:**1031-1034.

42. de Vries JJ, Baas WH, van der PK, Heesink A, Degener JE, Arends JP: **Outbreak of Serratia marcescens colonization and infection traced to a healthcare worker with long-term carriage on the hands.** *Infect Control Hosp Epidemiol* 2006, **27:**1153-1158.

43. Dineen P, Drusin L: **Epidemics of postoperative wound infections associated with hair carriers.** *Lancet* 1973, **2:**1157-1159.

44. Drusin LM, Ross BG, Rhodes KH, Krauss AN, Scott RA: **Nosocomial ringworm in a neonatal intensive care unit: a nurse and her cat.** *Infect Control Hosp Epidemiol* 2000, **21:**605-607.

45. Ejlertsen T, Prag J, Pettersson E, Holmskov A: **A 7-month outbreak of relapsing postpartum group A streptococcal infections linked to a nurse with atopic dermatitis.** *Scand J Infect Dis* 2001, **33:**734-737.

46. Elward A, Grim A, Schroeder P, Kieffer P, Sellenriek P, Ferrett R, Adams HC, Phillips V, Bartow R, Mays D, Lawrence S, Seed P, Holzmann-Pazgal G, Polish L, Leet T, Fraser V: **Outbreak of Salmonella javiana infection at a children's hospital.** *Infect Control Hosp Epidemiol* 2006, **27:**586-592.

47. Emberger M, Koller J, Laimer M, Hell M, Oender K, Trost A, Maass M, Witte W, Hintner H, Lechner AM: **Nosocomial Staphylococcal scalded skin syndrome caused by intra-articular injection.** *J Eur Acad Dermatol Venereol* 2011, **25:**227-231.

48. Esteban JI, Gomez J, Martell M, Cabot B, Quer J, Camps J, Gonzalez A, Otero T, Moya A, Esteban R: **Transmission of hepatitis C virus by a cardiac surgeon.** *N Engl J Med* 1996, **334:**555-560.

49. Faden HS, Burke JP, Glasgow LA, Everett JR, III: **Nursery outbreak of scalded-skin syndrome. Scarlatiniform rash due to phage group I Staphylococcus aureus.** *Am J Dis Child* 1976, **130:**265-268.

50. Faibis F, Laporte C, Fiacre A, Delisse C, Lina G, Demachy MC, Botterel F: **An outbreak of methicillin-resistant Staphylococcus aureus surgical-site infections initiated by a healthcare worker with chronic sinusitis.** *Infect Control Hosp Epidemiol* 2005, **26:**213-215.

51. Falcao DP, Mendonca CP, Scrassolo A, De Almeida BB, Hart L, Farmer LH, Farmer JJ, III: **Nursery outbreak of severe diarrhoea due to multiple strains of Pseudomonas aeruginosa.** *Lancet* 1972, **2:**38-40.

52. Felkner M, Pascoe N, Shupe-Ricksecker K, Goodman E: **The wound care team: a new source of group a streptococcal nosocomial transmission.** *Infect Control Hosp Epidemiol* 2005, **26:**462-465.

53. Finkelstein R, Reinhertz G, Hashman N, Merzbach D: **Outbreak of Candida tropicalis fungemia in a neonatal intensive care unit.** *Infect Control Hosp Epidemiol* 1993, **14:**587-590.

54. Foca M, Jakob K, Whittier S, Della LP, Factor S, Rubenstein D, Saiman L: **Endemic Pseudomonas aeruginosa infection in a neonatal intensive care unit.** *N Engl J Med* 2000, **343:**695-700.

55. Gaillard T, Gaillard C, Martinaud C, Vedy S, Pons S, Brisou P: **Epidemic surgical site infections attributable to incorrect use of face masks.** *J Hosp Infect* 2009, **71:**192-193.

56. Garibaldi RA, Rasmussen CM, Holmes AW, Gregg MB: **Hospital-acquired serum hepatitis. Report of an outbreak.** *JAMA* 1972, **219:**1577-1580.

57. Gaynes R, Marosok R, Mowry-Hanley J, Laughlin C, Foley K, Friedman C, Kirsh M: **Mediastinitis following coronary artery bypass surgery: a 3-year review.** *J Infect Dis* 1991, **163:**117-121.

58. Gould FK, Freeman R, Sisson PR, Cookson BD, Lightfoot NF: **Inter-strain comparison by pyrolysis mass spectrometry in the investigation of Staphylococcus aureus nosocomial infection.** *J Hosp Infect* 1991, **19:**41-48.

59. Gryska PF, O'Dea AE: **Postoperative streptococcal wound infection. The anatomy of an epidemic.** *JAMA* 1970, **213:**1189-1191.

60. Gupta A, Della-Latta P, Todd B, San Gabriel P, Haas J, Wu F, Rubenstein D, Saiman L: **Outbreak of extended-spectrum beta-lactamase-producing Klebsiella pneumoniae in a neonatal intensive care unit linked to artificial nails.** *Infect Control Hosp Epidemiol* 2004, **25:**210-215.

61. Haerem JW, Siebke JC, Ulstrup J, Geiran O, Helle I: **HBsAG transmission from a cardiac surgeon incubating hepatitis B resulting in chronic antigenemia in four patients.** *Acta Med Scand* 1981, **210:**389-392.

62. Harpaz R, Von Seidlein L, Averhoff FM, Tormey MP, Sinha SD, Kotsopoulou K, Lambert SB, Robertson BH, Cherry JD, Shapiro CN: **Transmission of hepatitis B virus to multiple patients from a surgeon without evidence of inadequate infection control.** *N Engl J Med* 1996, **334:**549-554.

63. Hedberg K, Ristinen TL, Soler JT, White KE, Hedberg CW, Osterholm MT, MacDonald KL: **Outbreak of erythromycin-resistant staphylococcal conjunctivitis in a newborn nursery.** *Pediatr Infect Dis J* 1990, **9:**268-273.

64. Hilton M, Chen JM, Barry C, Vearncombe M, Simor A: **Deoxyribonucleic acid fingerprinting in an outbreak of Staphylococcus aureus intracranial infection after neurotologic surgery.** *Otol Neurotol* 2002, **23:**550-554.

65. Hsu RB, Chen ML, Chang SC, Ko WJ, Chou NK, Wang SS, Chu SH: **Perfusionist-transmitted bacterial mediastinitis in a heart transplant recipient.** *Tex Heart Inst J* 2001, **28:**60-62.

66. Huang YC, Lin TY, Peng HL, Wu JH, Chang HY, Leu HS: **Outbreak of Candida albicans fungaemia in a neonatal intensive care unit.** *Scand J Infect Dis* 1998, **30:**137-142.

67. Isenberg HD, Tucci V, Cintron F, Singer C, Weinstein GS, Tyras DH: **Single-source outbreak of Candida tropicalis complicating coronary bypass surgery.** *J Clin Microbiol* 1989, **27:**2426-2428.

68. Jensenius M, Ringertz SH, Berild D, Bell H, Espinoza R, Grinde B: **Prolonged nosocomial outbreak of hepatitis A arising from an alcoholic with pneumonia.** *Scand J Infect Dis* 1998, **30:**119-123.

69. Johnston BL, MacDonald S, Lee S, LeBlanc JC, Gross M, Schlech WF, Chaudhary R, Langille D: **Nosocomial hepatitis B associated with orthopedic surgery--Nova Scotia.** *Can Commun Dis Rep* 1992, **18:**89-90.

70. Jones MR, Martin DR: **Outbreak of methicillin-resistant Staphylococcus aureus infection in a New Zealand hospital.** *N Z Med J* 1987, **100:**369-373.

71. Khuri-Bulos NA, Abu KM, Shehabi A, Shami K: **Foodhandler-associated Salmonella outbreak in a university hospital despite routine surveillance cultures of kitchen employees.** *Infect Control Hosp Epidemiol* 1994, **15:**311-314.

72. Klausner JD, Zukerman C, Limaye AP, Corey L: **Outbreak of Stenotrophomonas maltophilia bacteremia among patients undergoing bone marrow transplantation: association with faulty replacement of handwashing soap.** *Infect Control Hosp Epidemiol* 1999, **20:**756-758.

73. Kluytmans J, van Leeuwen W, Goessens W, Hollis R, Messer S, Herwaldt L, Bruining H, Heck M, Rost J, van Leeuwen N, .: **Food-initiated outbreak of methicillin-resistant Staphylococcus aureus analyzed by pheno- and genotyping.** *J Clin Microbiol* 1995, **33:**1121-1128.

74. Kolmos HJ, Svendsen RN, Nielsen SV: **The surgical team as a source of postoperative wound infections caused by Streptococcus pyogenes.** *J Hosp Infect* 1997, **35:**207-214.

75. Kurt TL, Yeager AS, Guenette S, Dunlop S: **Spread of pertussis by hospital staff.** *JAMA* 1972, **221:**264-267.

76. Lessing MP, Jordens JZ, Bowler IC: **Molecular epidemiology of a multiple strain outbreak of methicillin-resistant Staphylococcus aureus amongst patients and staff.** *J Hosp Infect* 1995, **31:**253-260.

77. Lettau LA, Smith JD, Williams D, Lundquist WD, Cruz F, Sikes RK, Hadler SC: **Transmission of hepatitis B with resultant restriction of surgical practice.** *JAMA* 1986, **255:**934-937.

78. Lo WT, Wang CC, Chu ML: **A nursery outbreak of Staphylococcus aureus pyoderma originating from a nurse with paronychia.** *Infect Control Hosp Epidemiol* 2002, **23:**153-155.

79. Locksley RM, Cohen ML, Quinn TC, Tompkins LS, Coyle MB, Kirihara JM, Counts GW: **Multiply antibiotic-resistant Staphylococcus aureus: introduction, transmission, and evolution of nosocomial infection.** *Ann Intern Med* 1982, **97:**317-324.

80. Lot F, Seguier JC, Fegueux S, Astagneau P, Simon P, Aggoune M, van Amerongen P, Ruch M, Cheron M, Brucker G, Desenclos JC, Drucker J: **Probable transmission of HIV from an orthopedic surgeon to a patient in France.** *Ann Intern Med* 1999, **130:**1-6.

81. Mastro TD, Farley TA, Elliott JA, Facklam RR, Perks JR, Hadler JL, Good RC, Spika JS: **An outbreak of surgical-wound infections due to group A streptococcus carried on the scalp.** *N Engl J Med* 1990, **323:**968-972.

82. McIntyre DM: **An epidemic of Streptococcus pyogenes puerperal and postoperative sepsis with an unusual carrier site--the anus.** *Am J Obstet Gynecol* 1968, **101:**308-314.

83. McKee WM, Di Caprio JM, Roberts CE, Jr., Sherris JC: **Anal carriage as the probable source of a streptococcal epidemic.** *Lancet* 1966, **2:**1007-1009.

84. McNeil SA, Nordstrom-Lerner L, Malani PN, Zervos M, Kauffman CA: **Outbreak of sternal surgical site infections due to Pseudomonas aeruginosa traced to a scrub nurse with onychomycosis.** *Clin Infect Dis* 2001, **33:**317-323.

85. Mean M, Mallaret MR, Andrini P, Recule C, Debillon T, Pavese P, Croize J: **A neonatal specialist with recurrent methicillin-resistant Staphylococcus aureus (MRSA) carriage implicated in the transmission of MRSA to newborns.** *Infect Control Hosp Epidemiol* 2007, **28:**625-628.

86. Mermel LA, McKay M, Dempsey J, Parenteau S: **Pseudomonas surgical-site infections linked to a healthcare worker with onychomycosis.** *Infect Control Hosp Epidemiol* 2003, **24:**749-752.

87. Mickelsen PA, Plorde JJ, Gordon KP, Hargiss C, McClure J, Schoenknecht FD, Condie F, Tenover FC, Tompkins LS: **Instability of antibiotic resistance in a strain of Staphylococcus epidermidis isolated from an outbreak of prosthetic valve endocarditis.** *J Infect Dis* 1985, **152:**50-58.

88. Molyneaux P, Reid TM, Collacott I, McIntyre PG, Dillon JF, Laing RB: **Acute hepatitis B in two patients transmitted from an e antigen negative cardiothoracic surgeon.** *Commun Dis Public Health* 2000, **3:**250-252.

89. Mossovitch M, Mossovitch B, Alkan M: **Nosocomial dermatophytosis caused by Microsporum canis in a newborn department.** *Infect Control* 1986, **7:**593-595.

90. Mutton KJ, Brady LM, Harkness JL: **Serratia cross-infection in an intensive therapy unit.** *J Hosp Infect* 1981, **2:**85-91.

91. Nahmias AJ, Godwin JT, Updyke EL, Hopkins WA: **Postsurgical staphylococcic infections. Outbreak traced to an individual carrying phase strains 80/81 and 80/81/52/52A.** *JAMA* 1960, **174:**1269-1275.

92. Naidoo J, Noble WC, Weissmann A, Dyke KG: **Gentamicin-resistant staphylococci: genetics of an outbreak in a dermatology department.** *J Hyg (Lond)* 1983, **91:**7-16.

93. Nakashima AK, Allen JR, Martone WJ, Plikaytis BD, Stover B, Cook LN, Wright SP: **Epidemic bullous impetigo in a nursery due to a nasal carrier of Staphylococcus aureus: role of epidemiology and control measures.** *Infect Control* 1984, **5:**326-331.

94. Occelli P, Blanie M, Sanchez R, Vigier D, Dauwalder O, Darwiche A, Provenzano B, Dumartin C, Parneix P, Venier AG: **Outbreak of staphylococcal bullous impetigo in a maternity ward linked to an asymptomatic healthcare worker.** *J Hosp Infect* 2007, **67:**264-270.

95. Okezie OA, Onyemelukwe NF: **Nosocomial infection in a Nigerian rural maternity centre: a series of nine cases.** *East Afr Med J* 2007, **84:**83-87.

96. Parry MF, Grant B, Yukna M, Adler-Klein D, McLeod GX, Taddonio R, Rosenstein C: **Candida osteomyelitis and diskitis after spinal surgery: an outbreak that implicates artificial nail use.** *Clin Infect Dis* 2001, **32:**352-357.

97. Parry MF, Hutchinson JH, Brown NA, Wu CH, Estreller L: **Gram-negative sepsis in neonates: a nursery outbreak due to hand carriage of Citrobacter diversus.** *Pediatrics* 1980, **65:**1105-1109.

98. Paul SM, Genese C, Spitalny K: **Postoperative group A beta-hemolytic Streptococcus outbreak with the pathogen traced to a member of a healthcare worker's household.** *Infect Control Hosp Epidemiol* 1990, **11:**643-646.

99. Payne RW: **Severe outbreak of surgical sepsis due to Staphylococcus aureus of unusual type and origin.** *Br Med J* 1967, **4:**17-20.

100. Pertowski CA, Baron RC, Lasker BA, Werner SB, Jarvis WR: **Nosocomial outbreak of Candida albicans sternal wound infections following cardiac surgery traced to a scrub nurse.** *J Infect Dis* 1995, **172:**817-822.

101. Pether JV, Caul EO: **An outbreak of food-borne gastroenteritis in two hospitals associated with a Norwalk-like virus.** *J Hyg (Lond)* 1983, **91:**343-350.

102. Petrosillo N, Raffaele B, Martini L, Nicastri E, Nurra G, Anzidei G, Ippolito G: **A nosocomial and occupational cluster of hepatitis A virus infection in a pediatric ward.** *Infect Control Hosp Epidemiol* 2002, **23:**343-345.

103. Prendergast TJ, Jr., Teitelbaum S, Peck B: **Transmission of hepatitis B by a surgeon.** *West J Med* 1991, **154:**353.

104. Prentice MB, Flower AJ, Morgan GM, Nicholson KG, Rana B, Firmin RK, Mitchell CJ: **Infection with hepatitis B virus after open heart surgery.** *BMJ* 1992, **304:**761-764.

105. Quinn RW, Hillman JW: **An epidemic of streptococcal wound infections.** *Arch Environ Health* 1965, **11:**28-33.

106. Rahav G, Pitlik S, Amitai Z, Lavy A, Blech M, Keller N, Smollan G, Lewis M, Zlotkin A: **An outbreak of Mycobacterium jacuzzii infection following insertion of breast implants.** *Clin Infect Dis* 2006, **43:**823-830.

107. Richards J, Williams H, Warner M, Johnson AP, Reith S, Woodford N, Marples RR, George RC: **Nosocomial spread of Staphylococcus aureus showing intermediate resistance to methicillin.** *J Hosp Infect* 1993, **25:**91-96.

108. Richardson JF, Quoraishi AH, Francis BJ, Marples RR: **Beta-lactamase-negative, methicillin-resistant Staphylococcus aureus in a newborn nursery: report of an outbreak and laboratory investigations.** *J Hosp Infect* 1990, **16:**109-121.

109. Richet HM, Craven PC, Brown JM, Lasker BA, Cox CD, McNeil MM, Tice AD, Jarvis WR, Tablan OC: **A cluster of Rhodococcus (Gordona) Bronchialis sternal-wound infections after coronary-artery bypass surgery.** *N Engl J Med* 1991, **324:**104-109.

110. Richman DD, Breton SJ, Goldman DA: **Scarlet fever and group A streptococcal surgical wound infection traced to an anal carrier.** *J Pediatr* 1977, **90:**387-390.

111. Roberts SA, Findlay R, Lang SD: **Investigation of an outbreak of multi-drug resistant Acinetobacter baumannii in an intensive care burns unit.** *J Hosp Infect* 2001, **48:**228-232.

112. Romano F, Ribera G, Giuliano M: **A study of a hospital cluster of systemic candidosis using DNA typing methods.** *Epidemiol Infect* 1994, **112:**393-398.

113. Ross RS, Viazov S, Gross T, Hofmann F, Seipp HM, Roggendorf M: **Transmission of hepatitis C virus from a patient to an anesthesiology assistant to five patients.** *N Engl J Med* 2000, **343:**1851-1854.

114. Roszczyk E, Kuchta A: **A new epidemic phage type of Staphylococcus aureus. II. Report on a hospital outbreak.** *Scand J Infect Dis* 1971, **3:**33-35.

115. Safdar N, Marx J, Meyer NA, Maki DG: **Effectiveness of preemptive barrier precautions in controlling nosocomial colonization and infection by methicillin-resistant Staphylococcus aureus in a burn unit.** *Am J Infect Control* 2006, **34:**476-483.

116. Saiman L, Cronquist A, Wu F, Zhou J, Rubenstein D, Eisner W, Kreiswirth BN, Della-Latta P: **An outbreak of methicillin-resistant Staphylococcus aureus in a neonatal intensive care unit.** *Infect Control Hosp Epidemiol* 2003, **24:**317-321.

117. Schaffner W, Lefkowitz LB, Jr., Goodman JS, Koenig MG: **Hospital outbreak of infections with group a streptococci traced to an asymptomatic anal carrier.** *N Engl J Med* 1969, **280:**1224-1225.

118. Schrack WD, Jr., Miller GB, Parkin WE, Fontana DB: **Four streptococcal infections traced to anal carrier.** *Pa Med* 1979, **82:**35-36.

119. Shanson DC, McSwiggan DA: **Operating theatre acquired infection with a gentamicin-resistant strain of Staphylococcus aureus: outbreaks in two hospitals attributable to one surgeon.** *J Hosp Infect* 1980, **1:**171-172.

120. Sheretz RJ, Reagan DR, Hampton KD, Robertson KL, Streed SA, Hoen HM, Thomas R, Gwaltney JM, Jr.: **A cloud adult: the Staphylococcus aureus-virus interaction revisited.** *Ann Intern Med* 1996, **124:**539-547.

121. Snider R, Landers S, Levy ML: **The ringworm riddle: an outbreak of Microsporum canis in the nursery.** *Pediatr Infect Dis J* 1993, **12:**145-148.

122. Snydman DR, Hindman SH, Wineland MD, Bryan JA, Maynard JE: **Nosocomial viral hepatitis B. A cluster among staff with subsequent transmission to patients.** *Ann Intern Med* 1976, **85:**573-577.

123. Spijkerman IJ, van Doorn LJ, Janssen MH, Wijkmans CJ, Bilkert-Mooiman MA, Coutinho RA, Weers-Pothoff G: **Transmission of hepatitis B virus from a surgeon to his patients during high-risk and low-risk surgical procedures during 4 years.** *Infect Control Hosp Epidemiol* 2002, **23:**306-312.

124. Stamm WE, Feeley JC, Facklam RR: **Wound infections due to group A streptococcus traced to a vaginal carrier.** *J Infect Dis* 1978, **138:**287-292.

125. Stein M, Navon-Venezia S, Chmelnitsky I, Kohelet D, Schwartz O, Agmon O, Somekh E: **An outbreak of new, nonmultidrug-resistant, methicillin-resistant Staphylococcus aureus strain (sccmec type iiia variant-1) in the neonatal intensive care unit transmitted by a staff member.** *Pediatr Infect Dis J* 2006, **25:**557-559.

126. Sundkvist T, Hamilton GR, Rimmer D, Evans BG, Teo CG: **Fatal outcome of transmission of hepatitis B from an e antigen negative surgeon.** *Commun Dis Public Health* 1998, **1:**48-50.

127. Sylvest B, Eriksen KR: **An outbreak of periporitis staphylogenes of complex origin.** *Acta Derm Venereol Suppl (Stockh)* 1979, **59:**181-184.

128. Syriopoulou VP, Hadjichristodoulou C, Daikos GL, Pirounaki M, Chatzicou V, Pavlopoulou I, Anagnostakou M, Theodoridou M, Dellagrammaticas H: **Clinical and epidemiological aspects of an enterovirus outbreak in a neonatal unit.** *J Hosp Infect* 2002, **51:**275-280.

129. Taneja N, Das A, Raman Rao DS, Jain N, Singh M, Sharma M: **Nosocomial outbreak of diarrhoea by enterotoxigenic Escherichia coli among preterm neonates in a tertiary care hospital in India: pitfalls in healthcare.** *J Hosp Infect* 2003, **53:**193-197.

130. Tanner EI, Bullin J, Bullin CH, Gamble DR: **An outbreak of post-operative sepsis due to a staphyloccoccal disperser.** *J Hyg (Lond)* 1980, **85:**219-225.

131. van den Broek PJ, Lampe AS, Berbee GA, Thompson J, Mouton RP: **Epidemic of prosthetic valve endocarditis caused by Staphylococcus epidermidis.** *Br Med J (Clin Res Ed)* 1985, **291:**949-950.

132. van Nierop WH, Duse AG, Stewart RG, Bilgeri YR, Koornhof HJ: **Molecular epidemiology of an outbreak of Enterobacter cloacae in the neonatal intensive care unit of a provincial hospital in Gauteng, South Africa.** *J Clin Microbiol* 1998, **36:**3085-3087.

133. Viglionese A, Nottebart VF, Bodman HA, Platt R: **Recurrent group A streptococcal carriage in a health care worker associated with widely separated nosocomial outbreaks.** *Am J Med* 1991, **91:**329S-333S.

134. Walsh B, Maguire H, Carrington D: **Outbreak of hepatitis B in an acupuncture clinic.** *Commun Dis Public Health* 1999, **2:**137-140.

135. Wang JT, Chang SC, Ko WJ, Chang YY, Chen ML, Pan HJ, Luh KT: **A hospital-acquired outbreak of methicillin-resistant Staphylococcus aureus infection initiated by a surgeon carrier.** *J Hosp Infect* 2001, **47:**104-109.

136. Welch J, Webster M, Tilzey AJ, Noah ND, Banatvala JE: **Hepatitis B infections after gynaecological surgery.** *Lancet* 1989, **1:**205-207.

137. Wenger PN, Brown JM, McNeil MM, Jarvis WR: **Nocardia farcinica sternotomy site infections in patients following open heart surgery.** *J Infect Dis* 1998, **178:**1539-1543.

138. Widmer AF, Wenzel RP, Trilla A, Bale MJ, Jones RN, Doebbeling BN: **Outbreak of Pseudomonas aeruginosa infections in a surgical intensive care unit: probable transmission via hands of a health care worker.** *Clin Infect Dis* 1993, **16:**372-376.

139. Zawacki A, O'Rourke E, Potter-Bynoe G, Macone A, Harbarth S, Goldmann D: **An outbreak of Pseudomonas aeruginosa pneumonia and bloodstream infection associated with intermittent otitis externa in a healthcare worker.** *Infect Control Hosp Epidemiol* 2004, **25:**1083-1089.

140. Zimmerman RA, Sciple GW: **Streptococcal wound infections.** *Rocky Mt Med J* 1966, **63:**63-65.
